# Paraneoplastic Neurological Syndromes: Study of Prevalence in a Province of the Lombardy Region, Italy

**DOI:** 10.3390/jcm9103105

**Published:** 2020-09-25

**Authors:** Lorenzo Lorusso, Vincenza Precone, Daniela Ferrari, Gaelle K. Ngonga, Antonio Giampiero Russo, Stefano Paolacci, Matteo Bertelli

**Affiliations:** 1 ASST Lecco, UOC Neurology and Stroke Unit, 23807 Merate (LC), Italy; l.lorusso@asst-lecco.it; 2 MAGI EUREGIO, 39100 Bolzano, Italy; vincenza_precone@yahoo.it (V.P.); matteo.bertelli@assomagi.org (M.B.); 3 Department of Neurology, Simone Veil Hospital, 95600 Eaubonne, France; ferrari.daniela76@gmail.com; 4 Department of Neurology, Hospital Centre Emile Mayrisch, Esch-sur-Alzette, 4240 Luxembourg, Luxembourg; ngonga.gaelle@yahoo.com; 5 UOC Unità di Epidemiologia, ATS della Città Metropolitana di Milano, 20122 Milan, Italy; agrusso@ats-milano.it; 6 MAGI’S LAB, 38068 Rovereto (TN), Italy; 7 EBTNA-LAB, 38068 Rovereto (TN), Italy

**Keywords:** paraneoplastic neurological syndrome, cancer, PNS prevalence, PNS epidemiology

## Abstract

Paraneoplastic neurological syndromes (PNSs) are a heterogeneous group of rare immune-mediated diseases associated with cancer. The aim of this study was to investigate the prevalence of PNSs in the province of Brescia. PNS prevalence was calculated using the Lombardy regional hospital admission records from 1998 to 2003. We used the website “Epidemiologic and Economic Atlas of Hospital Activities in Lombardy” and the “International Statistical Classification of Diseases and Related Health Problems”. In the province of Brescia, we found 54 cases of PNSs, 29 with subacute neuropathies, five with paraneoplastic cerebellar degeneration and 20 with encephalomyelitis. Peripheral nervous system diseases were the most frequent neurological disorders. In Lombardy, the number of PNS patients admitted was 322 (133 with encephalomyelitis, 21 with paraneoplastic cerebellar degeneration, 166 with polyneuropathies and two with optic degeneration). In Lombardy, the prevalence of PNSs was 25 in 100,000 hospital admissions and 5.92 in 100,000 for the Lombardy population. Our results show a discrete presence of PNS patients in the province of Brescia and in the Lombardy region as a whole.

## 1. Introduction

Paraneoplastic neurological syndromes (PNSs) are rare immune-mediated disorders caused by remote action of tumors in the absence of metastases, infection, ischemia or metabolic disruption [1]. PNSs may involve any part of the central and peripheral nervous system [2]. In 50–80% of patients, the neurological disorder develops before the cancer [3]. The diagnostic criteria of PNSs include manifestation of the typical neurological signs and detection of onconeural antibodies associated with cancer [4]. PNSs may be rapidly progressive and generally leave the patient severely debilitated. They may be associated with substantial morbidity [5]. Alternatively, they may have a progressive subacute clinical course, slow progression, relapses or a benign course [1]. Paraneoplastic Neurologic Syndrome Euronetwork guideline criteria are used internationally for the diagnosis and classification of “possible” and “definite” PNSs [5]. Treatment of PNSs includes treatment of the cancer, immunotherapy and supportive therapy [6]. It is important to discover PNSs because diagnosis can drastically change the prognosis and survival of cancer patients [5,7]. In fact, diagnosis and treatment of the tumor improves or stabilizes the clinical phenotype of PNSs [8]. The impact of PNSs on cancer prognosis is complex. A diagnosis of a PNS may lead to a diagnosis of cancer that would otherwise have remained hidden. It is also important to identify the cancer after diagnosis of the PNS to enable early diagnostic laboratory and imaging procedures and therapy.

The aim of the present study was to investigate the prevalence of PNSs, based on clinical neurological manifestations associated with tumors, in the province of Brescia, and to compare it with that of the Lombardy region as a whole.

Herein we seek to demonstrate that PNSs are widespread and already recognized in secondary care. The rarity of PNSs may depend on the difficulty of collecting a large group of PNS patients in centers equipped to conduct epidemiological studies. The few epidemiological studies on PNSs concern the incidence of specific syndromes [9,10], while other studies refer to the incidence in tertiary referral centers [3,11,12]. Prevalence studies are rarer and again refer to tertiary care [11] or to a specific PNS [13,14].

Paraneoplastic neurological syndromes are rare disorders that occur in less than 0.01% of patients with cancer [1,3]. Little epidemiological data are available on PNSs and secondary care or specific PNSs in cancer patients [3,11], although their incidence and prevalence are presumably not negligible. The choice of the Lombardy region and in particular the province of Brescia is motivated by the fact that the number of cancer patients and the prevalence of cancer in this area are the highest in Italy [15,16]. The reason for the high numbers is linked to high population density, whereas the reason for the high prevalence of tumors may be explained by socio-economic factors, as well as the concentration of economic activities such as industry, especially in the province of Brescia [17]. The Lombardy region has the highest population density in Italy and the province of Brescia is the largest in Lombardy, displaying a variety of socio-economic characteristics. The study of PNSs in cancer patients is particularly important because diagnosis of these syndromes can greatly improve prognosis and survival. Early diagnosis of PNSs can improve the treatment of tumors in secondary care [18].

## 2. Materials and Methods

### 2.1. Study Population

The prevalence of PNSs in the province of Brescia was retrieved from admission records. We used Lombardy regional hospital admission record codes, comparing those of the province of Brescia and Lombardy as a whole. The analysis was performed on the database created for PNS prevalence studies in the province of Brescia. The data were collected from a period of six years (1998–2003). The research used the website “Epidemiological and Economical Lombard Atlas of Hospital Activity” (www.aleeao.it). The hospital discharge form (in italian scheda di dimissione ospedaliera - SDO) codes for neurological syndromes and the main systemic cancers associated with them were retrieved from the International Statistical Classification of Diseases and Related Health Problems, 9th Revision—Clinical Modification (ICD-9—CM) of the World Health Organization (WHO) [19,20]. The prevalence of PNSs was estimated from the number of regional admission record codes of the public and private hospitals in the province of Brescia.

The data were collected by medical staff/doctoral students with study/research grants from the European project on paraneoplastic neurological syndromes. On the basis of the criteria of the Paraneoplastic Neurologic Syndrome Euronetwork guidelines [5], PNS patients were identified according to clinical presentation with one of the following neurological syndromes:

- cerebellar degeneration [21];
- encephalomyelitis, or limbic encephalitis [22];
- polyneuropathy [23];
- retinal degeneration [24];
- motor neuron disease [25];
- opsoclonus-myoclonus [26];
- peripheral nervous disorders, including sensory neuro-/neuronopathy, dysautonomia and neuromyotony [27]; and
- Lambert-Eaton myasthenia syndrome, polymyositis and necrotizing myelopathy [28,29].

Demographic data, clinical presentation, history of malignancy and related clinical testing including MRI brain, EEG and EMG/nerve conduction studies and comprehensive laboratory testing for suspected PNSs were recorded for all patients.

### 2.2. Statistical Analysis

Differences in the distribution of patient characteristics between groups were evaluated by analysis of variance and Chi-square analysis or a Student’s two-tailed *t*-test for continuous and categorical variables, respectively. A *p*-value less than 0.05 was considered statistically significant. All *p*-values were two-tailed. Data processing was carried out using IBM SPSS Statistics for Windows, Version 25.0, 2017 (Armonk, NY: IBM Corp).

## 3. Results

### 3.1. Epidemiological Data for the Lombardy Region

Table 1 shows the cases of PNSs and associated tumors in the Lombardy region from 1998 to 2003. The number of PNS patients admitted to hospitals was 322 (18 with encephalomyelitis, 115 with degenerative brain diseases, 21 with cerebellar degeneration, 166 with polyneuropathies and two with optic degeneration). There was a predominance of males (*n* = 222/69%).

Degenerative brain diseases and polyneuropathy were the most frequent neurological disorders detected in Lombardy (41% and 51%). While polyneuropathies prevailed in the male population (61%, *p* < 0.0001), there was a prevalence of degenerative brain diseases (49%) in the female population (*p* < 0.0001).

There was no statistical significant association between small cell lung cancer (SCLC) and degenerative brain diseases (*p* = 0.87), but there were significantly more male patients with SCLC (84%) and more female patients with breast cancer (55%) (*p* = 0.00001 for both).

In the Lombardy region the prevalence of PNSs was 25 in 100,000 hospital admissions and 5.92 in 100,000 of the Lombard population.

### 3.2. Epidemiological Data for the Province of Brescia

Table 2 shows the main characteristics of PNS patients admitted to hospitals in the province of Brescia between 1998 and 2003. The number of patients with PNS admission codes was 54, including 29 with polyneuropathies associated with SCLC, five with cerebellar degeneration (four with SCLC and one with colon cancer), one with encephalomyelitis associated with SCLC and 19 with degenerative brain diseases (dementia or extrapyramidal disorders) (14 with SCLC, three with breast cancer and two with colon cancer). In the province of Brescia, degenerative brain diseases and polyneuropathies were the most common neurological disorders, which was also true for the rest of Lombardy (*p* = 0.002), in both males and females (*p* = 0.67). Similarly, in the province of Brescia, SCLC was the cancer most associated with PNSs in males and females (*p* = 0.65).

The prevalence of PNSs was 30.54 in 100,000 hospital admissions and 8.33 in 100,000 for the population of Brescia.

## 4. Discussion

Paraneoplastic neurological syndromes are rare and have heterogenous neurologic clinical features [18,30]. The little epidemiological data available in the literature [11] mainly concern oncology and “secondary care”, indicating a frequency of about 0.01–8% in oncology patients and a high incidence of PNSs associated with SCLC, gynecological tumors and blood disorders [9,21]. Research on these diseases is therefore very important, because, if detected early, PNSs can be treated in cancer patients, increasing life expectancy and reducing medical costs [31,32].

The aim of this multicentric study was to determine the prevalence of PNSs in secondary care in the province of Brescia and compare it with the figures for the rest of Lombardy. Our preliminary results show a distinct presence of PNS patients in the province of Brescia and in Lombardy as a whole. The types of cancer that may induce PNSs are SCLC, breast, gastrointestinal tract and prostate cancer, which is in partial agreement with a recent retrospective study by Vogrig and colleagues, who found an association among lung, breast, lymphoma, gastrointestinal, ovary and urinary tract cancers in the provinces of Udine, Pordenone and Gorizia (but not Trieste) of the Friuli-Venezia Giulia region [11]. These provinces differ from Lombardy in socio-economic features. In Lombardy, 322 patients with PNSs were admitted to hospital (18 with encephalomyelitis, 115 with degenerative brain diseases, 21 with cerebellar degeneration, 166 with polyneuropathies and two with retinal degeneration). These results are different from those of studies by Vogrig et al. and Chan et al., who found limbic encephalitis, cerebellar degeneration and encephalomyelitis with greater involvement of the central than the peripheral nervous system, while Chandler et al. in the United Kingdom found clinical phenotypes similar to those reported in Lombardy [33]. This is probably due to the fact that Lombardy and the United Kingdom have overlapping socio-economic features, although caution is warranted in this comparison. In Lombardy, degenerative brain diseases are associated with breast cancer, as was reported in German patients by Finke et al. [14]. In our study, we found a predominance of males (*n* = 222; 69%), while in other studies a greater involvement of women, around 52%, has been reported [9,11]. PNS patients admitted to hospitals in the province of Brescia numbered 54 and included 29 with polyneuropathies associated with SCLC, five with cerebellar degeneration (four with SCLC and one with colon cancer), one patient with encephalomyelitis and SCLC and 19 patients with degenerative brain diseases (14 with SCLC, three with breast cancer and two without tumors). In line with previous studies, prevalence analysis for the province of Brescia and the region of Lombardy confirmed that degenerative brain diseases and polyneuropathies are the most frequent PNSs [3,5,9,11,12,34].

Most cases (34 of 53) were detected in the five hospitals of the city of Brescia. This may be due to a better and more efficient diagnostic process. Interestingly, a proportion of the other cases were detected in hospitals of the Brescia lakes area (lakes Iseo and Garda).

At the regional level, we recorded a prevalence of 25 in 100,000 hospitalizations and 5.92 cases per 100,000 of population. The prevalence in Brescia province was 30.54 in 100,000 hospital admissions and 8.33 in 100,000 for the population of Brescia. Our epidemiological data over six years in Lombardy therefore showed a higher prevalence of PNSs than reported in the literature, especially when the data are compared with that of the Friuli-Venezia Giulia region (prevalence: 4 in 100,000) reported by Vogrig et al. [2,3,11,35]. The higher prevalence in Lombardy is due to socio-economic differences and the greater industrialization of Lombardy and the province of Brescia.

Our study has some limitations. The results are not accurate because the codes used are not specific to PNSs (specificity: 50%), and may therefore have been overestimated. There is also no information on associated autoantibodies, because there was no code for their identification and associated therapy. For example, the frequency of neuropathies could be distorted by their frequent association with metabolic neuropathies, prostate cancer in the elderly and side effects of therapies, and the same is true of degenerative brain disorders where we cannot distinguish dementia from extrapyramidal disorders. Likewise, there may have been an underestimation of data in cases where a tumor was detected after diagnosis of a PNS, due to early manifestation of the neurological disorder, lack of diagnostic specificity and sensitivity or underestimation or failure to detect neurological symptoms. However, we took certain steps to ensure accurate estimation of the results: International criteria for diagnostic classification of patients were applied and only “definite” PNS patients were considered. We also used Graus criteria [34], which do not require antibody positivity [5,36]. The results confirm the rarity of PNS in line with European indications but they also show that the prevalence is not negligible. Healthcare workers therefore need to pay greater attention to identifying PNSs. It is also fundamental to know the magnitude of PNS, since its incidence is similar to that of infectious encephalitis [37].

Paraneoplastic neurological syndromes may affect the central or peripheral nervous systems [1,37]. Our data showed that peripheral nervous system involvement was more frequent (80%), whereas degenerative brain diseases (dementia + extrapyramidal disorders) are the most frequent PNSs.

Notwithstanding these limitations, our findings add important elements to our understanding of the epidemiology of PNSs, highlighting the fact that doctors bear these syndromes in mind, despite their rarity, and make diagnoses, as demonstrated by our study. More epidemiological data on PNSs could promote awareness of these syndromes among health operators, enabling oncology units to take a preventive diagnostic and therapeutic approach. To our knowledge there have been no long-term follow-up studies of PNS patients with cancer. We conclude that these neurological disorders associated with cancer warrant more attention.

## Figures and Tables

**Table 1:** Cases of pareneoplastic neurological syndromesassociated with tumors in the Lombardy region from 1998 to 2003. f = female, m = male.

Neurological Phenotype	Sex			Associated Tumors			
	Female *n* (%)	Male *n* (%)	Total *n* (%)	Lung Cancer	Breast Cancer	Colon Cancer	Gynecological Cancer
Encephalomyelitis	9 (9%)	9 (4.1%)	18 (5.6%)	11 (2 f, 9 m)	5 f	2 f	/
Degenerative brain diseases (dementia + extrapyramidal disorders)	49 (49%)	66 (29.7%)	115 (35.7%)	68 (14 f, 54 m)	29 f	18 (6 f, 12 m)	
Cerebellar degeneration	12 (12%)	9 (4%)	21 (6.5%)	10 (2 f, 8 m)	9 f	1 m	1 f
Polyneuropathy	30 (30%)	136 (61.3%)	166 (51.6%)	166 (30 f, 136 m)	/	/	/
Retinal degeneration	0	2 (0.9%)	2 (0.6%)	2 m	/	/	/
Opsoclonus-myoclonus	0	0	0	/	/	/	/
Total	**100**	**222**	**322**	**257**	**43**	**21**	**1**

**Table 2:** Distribution of PNS patients in hospitals in the province of Brescia from 1998 to 2003. f = female, m = male.

Neurological Phenotype	Sex			Associated Tumors			
	Female *n* (%)	Male *n* (%)	Total *n* (%)	Lung Cancer	Breast Cancer	Colon Cancer	Gynecological Cancer
Encephalomyelitis	1 (6%)	0	1 (2%)	1 f	/	/	/
Degenerative brain diseases (dementia + extrapyramidal disorders)	10 (59%)	9 (24%)	19 (35%)	14 (6 f, 8 m)	3 f	2 (1 f, 1 m)	/
Cerebellar degeneration	0 (0%)	5 (14%)	5 (9%)	4 m	/	1 m	/
Polyneuropathy	6 (35%)	23 (62%)	29 (54%)	29 (6 f, 23 m)	/	/	/
Retinal degeneration	0	0	0	/	/	/	/
Opsoclonus-myoclonus	0	0	0	/	/	/	/
Total	**17**	**37**	**54**	**48**	**3**	**3**	**0**
